# A Survey of Attitudes and Beliefs about Care, Compassion and Communities Networks in Palliative Care. A Preliminary Study for the Development of a Compassionate University

**DOI:** 10.3390/healthcare9080946

**Published:** 2021-07-27

**Authors:** Silvia Librada Flores, Sonia Herminia Roa Trujillo, Nurlian Torrejano Gonzálex, María del Pilar García Buitrago, Miguel Ángel Lucas Díaz

**Affiliations:** 1 New Health Foundation, 41014 Sevilla, Spain; miguelangel.lucas@newhealthfoundation.org; 2 Welfare Department, Sanitas University Foundation, Bogota 111321, Colombia; shroa@unisanitas.edu.co (S.H.R.T.); unisanitascompasiva@unisanitas.edu.co (N.T.G.); mpgarcia@unisanitas.edu.co (M.d.P.G.B.)

**Keywords:** palliative care, Compassionate University, empathy, community networks, student health services, education

## Abstract

The aim of this study was to know the level of knowledge, sensitivities and training needs regarding care of people at the end of life in medicine, nursing and psychology students/academic and administration university personnel; and to identify skills to perceive and expressed values related to compassion it in their living environment. Method: a descriptive observational study was conducted among undergraduate medical, nursing and psychologist students, academic and administration personnel of the University of Bogotá in Colombia the survey was based on a web-based questionnaire (November 2019–April 2020). Levels of knowledge and sensitivities about care of people at the end of life, educational needs and compassion were assessed. Descriptive and comparative measures and statistical significance tests used, Student’s t and ANOVA (α = 0.05). Results: 465 people answered the survey; students (82.4%), academic (13.1%) and administration personnel (4.5%). 81.6% knew about palliative care concepts. 64.7% had not cared for other people with advanced or terminal illness. 44.7% talked about death without problems. The most evaluated training competences were humanity, dignity and compassion. Mean levels for compassion by Gilbert’s scale were 70.55 for self-compassion, 72.61 for compassion for others and 60.47 for compassion from others. Significant differences were found by age and gender in self-compassion values. Conclusions: the level of knowledge, sensitivities and training needs regarding care of people at the end of life in the University and the values related to compassion enables the development of Compassionate Universities.

## 1. Introduction

Care, compassion and community are considered essential elements for the care of people with advanced disease and/or at the end of life and it’s necessary to incorporate these concepts progressively in the Universities that also affect the quadruple aim in health from the benefits of compassion: patients´ benefits, population health, professional’s wellbeing’s and effective organizations. [1,2]. As expressed by Lown et al. [3] “care without compassion cannot be provided and compassion without an element of empathy and help towards the other cannot be well applied”. Community involvement is an essential element for person-centered care where care can be redistributed among a range of members involved in care [4].

University is an institution that seeks to generate a series of competencies towards the best practices of professional development and in its relationship with people. In recent years, training in palliative care has been gradually implemented in Universities in Latin America [5]. Among the main topics included in the curricular proposals for the training of doctors, nurses and psychologists in Palliative Care (PC) are: (1) basics concepts of PC, (2) pain and symptom management, (3) psychosocial and spiritual aspects, (4) ethical and legal issues, (5) communication and (6) teamwork and self-reflection [6]. Being fundamental elements of the curriculum, there is a lack of knowledge about students and professionals´ perceptions about palliative care, its relationship with death, its will to care for someone close to them or if they have skills to be empathetic or compassionate with people around them [5].

A “Compassionate University” is an organization that is committed to developing and facilitating the practice of compassion in students and health professionals for the creation of more humane, dignified and compassionate health systems.

“Compassion” can be defined as a sensitivity to the suffering of self and others with a commitment to prevent it and relieve it. As a complex and multifaceted response to suffering, compassion involves sensitivity, recognition, understanding, emotional resonance, empathic concern and distress tolerance for another’s pain or suffering, coupled with motivation and relational action to ameliorate it [2]. So, as starting point, it is necessary to identify how each of us relates to care, compassion and community involvement. The best way to build ourselves as compassionate beings is to have our own experience that we are going to die, that we are going to need to be cared for and that surely in our lives we are going to have to take care of a relative or some other close person.

A recent study carried out in Ecuador has shown that the identification of these factors and the actions implemented to promote compassion in the university and create a compassionate university have been beneficial in terms of greater satisfaction of students and teachers thanks to the skills and values acquired during this stage at the University [7].

From this perspective, it is essential to offer students and professionals the best tools and skills to provide this quality care, incorporating these concepts into the curriculum and developing awareness-raising actions towards care that are spread throughout the educational community.

The Sanitas University Foundation of Bogotá, Colombia is committed not only to the quality of teaching, but also to the dignity of people, humanization and compassion. Thus they decided to launch a project of “Compassionate University” together with the New Health Foundation, which has its own methodology for the development of Compassionate Communities (All with you^®^ method) [8] which is also being applied to Universities with the main objective of building a University that recognize for its culture of cultivating empathy, compassion and caring for people who face difficult situations inside and outside the organization, as well as fostering the development of community networks at the University to help from within and without those who are with a situation of advanced disease and/or at the end of life.

Based on this objective of becoming a Compassionate University, a preliminary study has been carried out with the purpose of:

- Identifying the level of knowledge and sensitivities that professionals and students have regarding the care and attention of people at the end of life.
- Detecting the training needs—according main topics included in the curricular proposals for the training of doctors, nurses and psychologists in Palliative Care-, within the university teaching programs related to the care of people with advanced disease and/or at the end of life.
- Identifying the abilities of professionals and students to perceive values related to compassion and express it in their life environment.

## 2. Materials and Methods

### 2.1. Study Design and Population

Descriptive observational study. A 63-item web-based questionnaire was design by New Health Foundation. An invitation letter with the link to the survey were emailed to all undergraduate medical, nursing and psychologist students from first to sixth year of education, academics and professionals of the University (*N* = 650). Data collection was carried out Since November 2019 to April 2020.

### 2.2. Variables and Measures

The survey was designed in 4 blocks:

- Block 1. Sociodemographic and academic characterization: sex, age, household structure, academic relationship with the University.
- Block 2. Level of knowledge and sensitivities of the population about care of people at the end of life.
- Block 3. Training needs of students related to care of people with advanced disease and/or at the end of life. The competencies were classified according to the European Association for Palliative Care (EAPC) curriculum [6] on PC in Universities.
- Block 4. Assessment of Compassion in students and professionals on self-compassion, compassion for others and compassion from others. The validated Gilbert´s scale was used [9].

### 2.3. Statistical Analysis

Descriptive and comparative frequency measures were used by blocks of contents of the survey and by type of profile of respondents. Student’s t-statistical significance tests and one-way ANOVA were performed to compare the mean values on the compassion scale in the distribution by sex, age and professional profile. An α value of 0.05 was established to determine statistical significance. The SPSS program was used for statistical analyzes.

All respondents agreed to the use and treatment of the data for the research.

### 2.4. Ethical Considerations

Participation and acceptance of the survey was requested through written consent for the use of the data for research purposes, guaranteeing the anonymity and confidentiality of the information to all participants. The study took into account the Declaration of Helsinki and resolution 008430 of the Ministry of Health [10]. The data used were for the exclusive use of the investigation and the identity of the individuals was protected according to Law 1581 [11].

## 3. Results

### 3.1. Sociodemographic and Academic Characterization of the Study Population

A total of 465 surveys corresponding to university students, academics and professionals of University were collected, representing a response rate of 71.5%. 74% women, 26% men. Mean age: 24 years (standard deviation: 10,003). 74.6% couple without children.

The highest participation in the surveys were students with a total of 383 participants (82.4%). 167 were nursing students (43.6%), 128 medical students (33.4%) and 77 psychology students (20.2%). The highest representation of students corresponded to first-year students (48.8% of cases), followed by third-year students (20.6%).

The university academics and others professionals were represented by 61 academics (13.1% of the population), of which 26.2% were nursing academics, 24.6% psychology academics and 23% medicine academics. The professional corresponding to administration represented 4.5% of the professionals. The Mean dedication of the academics at the university was 4.5 years. The sociodemographic and academic characterization of study population is represented in Table 1.

### 3.2. Level of Knowledge and Sensitivities of the Population about Care of People at the End of Life 

363 people (81.6%) knew palliative care concepts, most of them as part of their university education or their profession (67.2%), followed by family members (13.6%), friends (9.1%), social media (8.8%), personal experience (7.4%) or other reasons (3.1%).

The people who reported knowing how to give a definition of palliative care associated this concept with the provision of palliative care for adults and pediatric population in a situation of chronic, advanced and/or end-of-life disease, to the decrease in suffering, improvement of the quality of life, state of agony and end of life, and the privilege of caring and being cared for people.

72.9% (339 people) reported having had previous contact with palliative care due to the experiences of patients or close relatives. Of these, 255 (77.3%) received palliative care. In 79.3% of the cases, they were valued as useful both for the person at the end of their life and for their family and their entire care network.

64.7% of the study population had not cared for people with advanced or terminal illness. 91.2% would be willing to take care of a person who was not a relative or close friend. 50.8% of those surveyed indicated that they felt capable of accompanying a person at the end of their life.

Among the 465 people who answered the question: “How many people do you think would be able to take care of you if an illness were to overtake you at this time in your life?” 67.5% (313) indicated that fewer than 5 people would be involved in their care, 24.6% (114) between 5 and 9 people and 7.9% (38) more than 10 people.

49.2% did not feel capable of accompanying a person at the end of their life. Those who indicated feeling capable were those who had received some training in CP.

44.7% of those surveyed (*n* = 208) referred to talking about death without a problem, 24.5% do so very rarely, 20.9% sometimes, but with people from the environment and professional profile, 6.7% when it occurs in a way close and 3.2% never.

The level of knowledge and sensitivities of the population about the care of people at the end of life is represented in Table 2.

### 3.3. Students Training Needs Related to Caring of People with Advanced Disease and/or at the End of Life

47.7% of the students reported not having previous training in palliative care. 52.3% whom had training in palliative care, 32.8% had attended it through training at the university, 8.7% through continuous training, 3.4% in postgraduate courses and 7.4% other training outside the university.

The highest training in Palliative Care at the University was received by nursing students (50%), followed by medical students (26.7%) and psychology students (13%).

Nursing students indicated that they would prefer to dedicate themselves professionally to PC (58.1%), followed by medicine (43%) and very closely by psychology (42.9%).

The subjects received in palliative care and the interest in receiving training in these subjects among the students of the different faculties and the professors are represented in the Table 3.

**Figure 1:**
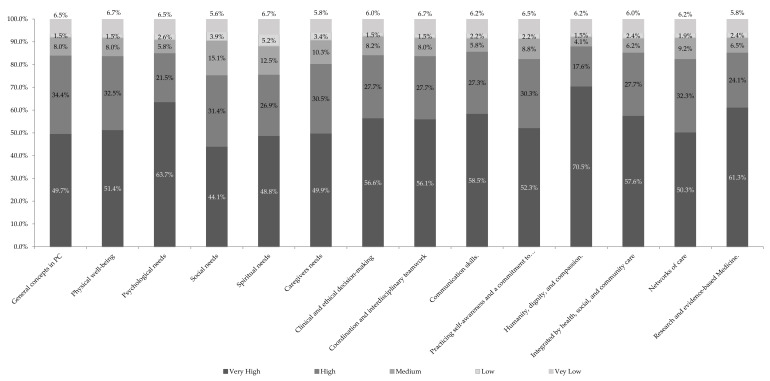
Priority level for competences to be developed in training related to Palliative Care.

The training competences most valued by students, academics and professionals of the university was Humanity, Dignity and Compassion, which was considered by 70.5% of those surveyed as a high priority. (Figure 1).

### 3.4. Self-Compassion, Compassion for Others, Compassion from Others; Gilbert’s Scale

For a total of 465 people who answered the compassion survey, a Mean value of 72.61 was obtained for compassion for others, 70.55 for self-compassion and 60.47 for compassion from others.

**Figure 2:**
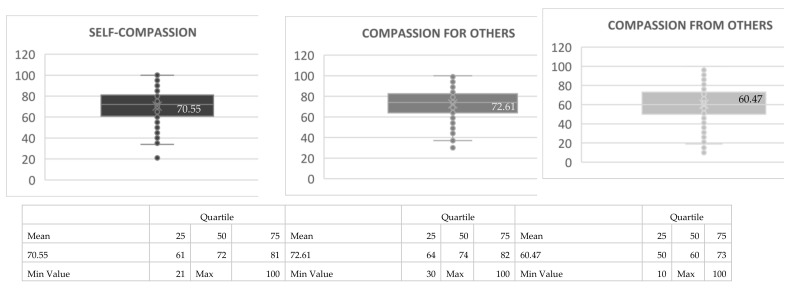
Gilbert´s compassion scale.

Mean values on the compassion scale are represented on the Figure 2.

Men scored higher on self-compassion (Mean 73.04), compassion for others (73) and compassion from others (61.30).

Self-compassion, compassion for others and compassion from others were most valued in people aged 60 and over.

Compassion for others was the most valued among psychology students (74.94), self-compassion the most valued among teachers (73.77) and compassion from others among nursing students (61.82).

Significant differences were found for gender and age in self-compassion values (*p* = 0.028, *p* = 0.039, respectively). No significant differences were obtained in the values of compassion by academic profile. The results of the compassion and *p*-values surveys are represented in Table 4.

## 4. Discussion

We offer some data on compassion and palliative care among university students, academics and other professionals that can help on improving the skills and self-awareness of future health care professionals [12]. One of two doctors and patients reports that care is not compassionate despite being a preferred element in the care and relief of suffering [13]. The practice of compassion is beneficial and even more so in the most vulnerable moments such as advanced disease and at the end of life. The benefits of compassion in PC have been evidenced by Brito and Librada [2], impacting the quadruple health goal: patients´ benefits, population health, professional’s wellbeing’s and effective organizations. In this way, the University is the most suitable environment to create compassionate professionals and leaders who act through a more humane, dignified and compassionate treatment in the care of people, especially at the end of life.

This study has been carried out with the objective of identifying the sensitivities and knowledge of the university community towards care, compassion and the community. The development of this baseline diagnosis in students, academics and professionals of the University it´s the first stages for the development of a Compassionate University. Results will allow designing actions at the University aimed at raising awareness, training and research in this field.

Training in Palliative Care is an essential component in the faculties of health sciences, even more so when death is a natural process of life that all people are going to encounter. According to Latin American Atlas of PC [14], only 30% of the Universities in Latin American countries teach these topics, and there is also a disproportion of the contents taught or teaching hours. In our study, the greatest knowledge about palliative care in students comes from studies at the University. Even so, up to 18.4% of those surveyed indicated that they did not know what palliative care is, associating this concept in a greater proportion with death, with the state of agony and care. Those who expressed having knowledge in PC, associated it with the decrease in suffering, the improvement of the quality of life, care and to a lesser extent with the state of agony and death [15].

91.2% of those surveyed would be willing to take care of a person who was not a relative or close friend, although up to 49.2% indicated that they would not feel capable of doing so. Sometimes half of the graduates do not feel prepared to attend the end of life as shown by the studies by Fraser et al. [16]. There are also references that up to 35% of medical students have not observed a patient at the end of life [13]. This can cause fear in students in the face of death due to the feeling of not having enough tools to deal with this situation. This question was deliberately asked to reflect later with the students in these analyzes and to emphasize that care should not be directly related to the profession, but to the willingness and commitment to help, which is an inherent condition of the human being.

In the same way, it is worth highlighting the answer to the question, how many people do you think would be able to take care of you if an illness were to overtake you at this point in your life? This question is being asked by the New Health Foundation to all types of people of all age groups and usually the Mean number of people who identify themselves is 4 people. In our population, up to 67.5% indicated that less than 5. The concept of care is usually related to the development of tasks related to the basic activities of daily life and with first and second degree people involved in care. In a study carried out on 99 terminally ill people, other profiles that may be involved in caring for people at the end of life were identified and that they can develop other types of tasks [17]. In this way, as Julian Abel expresses in his model of care circle [4], it is necessary to sensitize the population to the presence of other profiles (friends, co-workers, neighbors, etc.) that can carry out tasks that are complementary to those of a main caregiver. The increase in these care networks improves the quality of life of the person, reduces the burden of the main caregiver and improves the satisfaction of patients and their families. These results are already being analyzed in a community intervention process through the RedCuida protocol for the creation and management of care networks [18,19].

Death is not entirely present in the Universities. 44.7% of those surveyed indicated that they talk about it without problem. However, professional practice should bring us closer to talking about death since experiences with patients can bring us closer to these sensitivities towards it. Not talking about death makes us not empathize with the death of the other, and this has caused health professionals frustrations on many occasions. In other studies, carried out on the approach to death of students, communication needs about death with close people, patients or children have also been identified [20]. It´s necessary to implement in the University themes about death and programs such as Death Café [21] in the university are being implemented along this line to bring together not only health sciences students, but also the rest of the university community.

The topics least covered in the faculties of medicine, psychology and nursing are Management and Organization, Public Policies, Pediatric Palliative Care, Network Management and Compassion. These results coincide with those of Billings et al. [22] in 1,455 medical students where the lack of communication and compassion aspects is expressed in the training curriculum in end of life care. There is a tendency to focus the topics on the most specialized areas of the profession, leaving vacant topics related to organization, management, research, death, emotional skills and values of humanization, compassion and dignity. Therefore, the results of this first study indicate that these areas must be reinforced in the curriculum and in the rest of the awareness-raising actions that are carried out at the university and that have to do with the development of the Compassionate University.

Humanization, dignity and compassion were the skills most valued by the students. 70.5% considered it a high priority for adequate care of people at the end of life. These results coincide with those of Borgstrom et al. [23], Centeno et al. [24] and Hurwitz et al. [25] that indicate the competences of dedicated time with patients, learning about wider elements of treatment and holistic care, communications skills and learning about themselves through reflective writing.

The Gilbert Compassion Scale [9] applied to students and professionals it values components of action and commitment of compassion towards oneself, towards others and the compassion that we receive from others. The survey has been applied with the aim of evaluating compassion on a personal level, although the students may have been conditioned in their responses according to the career they were studying or their personal relationship with palliative care.

The results are remarkable in each one of the blocks, being the least valued the one of compassion of the others. In the interpretations made later at the University with the professors and students about these answers, it was concluded that we usually relate more with the help to the other than with the help we receive from the other. As expressed by Brito et al. [2], the benefits of compassion in palliative care can bring us closer to improving care for people at the end of life.

The results of this first diagnosis at the University coincide with those of Dávalos et al. [7], where the same research was carried out within the framework of the Compassionate University for a sample of 459 students and 77 members of the University. The development of this line of research is allowing Universities to advance in response to a series of needs and motivations in students and professors: there is a willingness to care, the values of compassion are notable in the students and professors of the faculties of health sciences and it is necessary to include more topics on care, compassion and the community as transversal axes of training in the curriculum.

This study has been carried out with the objective of knowing the sensitivities and knowledge towards the end of life at a personal rather than an academic level and from here to propose a training curriculum together with a series of complementary actions in the development of a Compassionate University. It integrates the elements of care, compassion and community to work from its analysis in the development of a Compassionate University based on its own methodology of diagnosis, research and action that is being applied to other universities in Spain and Latin America. Thanks to the methodology and the results that are extracted from this study, the development of a Compassionate University is allowed since the surveys and analyzes of training needs regarding care, compassion and the community allow the development of initiatives that make a Compassionate University.

## 5. Limitations

The surveys that were carried out in this first diagnosis were carried out anonymously, so it has not been possible to make a comparison before and after launching a series of actions at the University that promote the values of care.

## 6. Conclusions

The development of this survey, which contains a high reflective component on care, compassion and the way we behave with our own environment at the end of life, has allowed students to approach the knowledge of the subject in a way closer and compassionate, mobilized by action and not considered as a theoretical subject.

Thanks to the results of this first diagnostic study, a Compassionate University project is being developed at the University that sensitizes, trains and mobilizes students and professionals to develop care networks around people at the end of life.

Compassion must be extended beyond professional competencies, making care for the people around us extend from the University.

## Figures and Tables

**Table 1:** Sociodemographic and academic characterization of the study population.

**Variables**					**Total Sample** ***N*: 465**		**%**
**Sociodemographic characterization**							
**Sex**							
Male					121		26%
Female					344		74%
**Age group**							
• 18–39 years					412		88.6%
• 40–59 years					49		10.5%
• Over 60 years					4		0.9%
**Mean age**					24		
**Standard deviation**					10,003		
**Household structure**							
Couple without children					347		74.6%
Married with children					29		6.2%
Single with children					25		5.4%
Cohabitant without children					22		4.7%
Cohabitant with children					17		3.7%
Married without children					14		3%
Separated with children					6		1.3%
Separated without children					5		1.1%
**Students**							
**Students *n* = 383 (82.4%)**	**Total students (%)**	**(Semester 1/2)**	**(Semester 3/4)**	**(Semester 5/6)**	**(Semester 7/8)**	**(Semester 9/10)**	**(Semester 11/12)**
Medicine Students	**128** (33.4%)	69 (53.8%)	24 (18.5%)	10 (7.6%)	8 (5.9%)	9 (6.7%)	10 (7.6%)
Psychology Students	**77** (20.2%)	51 (65.8%)	10 (13.2%)	9 (11.8%)	7 (9.2%)	0 (0.0%)	0 (0.0%)
Nursing Students	**167** (43.6%)	62 (37.1%)	27 (16.2%)	58 (34.7%)	20 (12.0%)	0 (0.0%)	0 (0.0%)
No specification	**11** (2.8%)						
Total	**383 (100%)**	181 (48.8%)	61 (16.3%)	77 (20.6%)	35 (9.3%)	9 (2.3%)	10 (2.6%)
**Teachers**						**Total Sample**	**%**
Professor of the University of Nursing						16	26.2%
Professor at the University of Psychology						15	24.6%
Professor at the University of Medicine						21	23.0%
No job specification						9	14.8%
**Total Teachers**						***n* = 61**	**13.1%**
**Other professionals at the University**							
Academic directors						7	33.3%
Operational area						5	23.9%
Academic support executive and authority area						4	19.0%
Administration area						3	14.3%
No job specification						2	9.5%
**Total Other University professionals**						*n* = 21	4.5%

**Table 2:** Level of knowledge and sensitivities of the population about the care of people at the end of life.

**Knowledge and Sensitivities towards Palliative Care**	**YES**						**NO**					
	**Medicine**	**Psychology**	**Nursing**	**Teachers**	**Other Professionals**	**Total**	**Medicine**	**Psychology**	**Nursing**	**Teachers**	**Other Professionals**	**Total**
Do you know what palliative care is? (*n*: 445)	110 30.3%	50 13.8%	139 38.3%	46 12.7%	18 5.0%	363 * **81.6%**	18 4.8%	27 7.1%	28 7.4%	6 1.6%	3 0.8%	82 ** **18.4%**
**Who Do You Consider They are Aimed at (*n*: 442)**			**Medicine**	**Psychology**		**Nursing**		**Teachers**		**Other Professionals**	**Total**	
- To the entire population (adult and pediatric population) with advanced disease and/or at the end of life			110 24.9%	62 14.0%		150 33.9%		47 10.6%		15 3.4%	**384** **86.9%**	
- Only to the adult population with advanced disease and/or at the end of life			17 3.8%	13 2.9%		16 3.6%		4 0.9%		2 0.5%	**52** **11.8%**	
- Only to the pediatric population with advanced disease and/or at the end of life			1 0.2%	2 0.5%		1 0.2%		1 0.2%		1 0.2%	**6** **1.4%**	
**Concepts Associated with Palliative Care (*n* = 1115 Multiple Responses)**												
			If you know palliative care (*n* = 950)					Does not know palliative care (*n* = 165)				
- Death			105 (11.1%)					22 (13.3%)				
- State of agony and end of life			164 (17.3%)					37 (22.4%)				
- Decrease in suffering			276 (29.1%)					41 (24.8%)				
- Quality of life			263 (27.7%)					38 (23%)				
- The privilege of caring and being cared for			138 (14.5%)					26 (15.8%)				
- Other concepts			4 (0.4%)					1 (0.6)				
**Experiences of Contact with People in Palliative Care**												
**Temporality**			**Yes = 339 (72.9%)**									**No = 126 (27.1%)**
			**Nowadays**		**In the last month**			In the last year		More than 1 year ago		
			**41 (12.1%)**		**27 (8.0%)**			97 (28.6%)		174 (51.3%)		
Relationship			Patient	Grandparent	Uncle	Father mother	Friend	Work partner	Sibling	Spouse/Partner	Other	
			95 (28.9%)	92 (28.0%)	52 (15.8%)	30 (9.1%)	25 (7.6%)	7 (2.1%)	4 (1.2%)	2 (0.6%)	22 (6.7%)	
**Received Palliative Care**												
Usefulness of CP	**Yes = 255 (77.3%)**											**No = 75 (22.7%)**
	Yes and I only considered it useful for the person who was going through this disease process			Yes, and I considered them useful both for the person with the disease, as well as for their family and their entire care network.			Yes and I considered them useful, but only for the family and their care network.			Do not consider them useful		
	36 (145.9%)			191 (79.3%)			4 (1.7%)			10 (4.1)		
**Experiences of Caring for Someone at the End of Life**												
Have cared for a person at the end of life		**Yes = 164 (35.3%)**										**No = 301 (64.7%)**
Hours of dedication to care Mean of 7 h of dedication		<6 h		6–11 h		12–17 h		18–23 h		24 h		
		41 (51.7%)		27 (23.1%)		(18.2%)		97 (1.4%)		(5.6%)		
People involved in care Mean of 8 people involved		<5 people		5–9 people		10–14 people		15–19 people		> 20 people		
		55 (41.4%)		52 (39.1%)		10 (7.5%)		5 (3.8%)		11 (8.3)		
**Willingness and Ability to Care**												
Willingness to take care of a person other than the closest family or circle of friends			**Si = 424 (91.2%)**					**No = 41 (8.8%)**				
You feel able to care for a person with advanced disease			**Yes = 236 (50.8%)**					**No: 229 (49.2%)**				
**Care Network (*n* = 465)**	**Less Than 5 People**		**Between 5 and 9 People**		**Between 10 and 14 People**		**Between 15 and 19 People**		**Between 20 and 24 People**		**More Than 25 People**	
	313 (67.5%)		114 (24.6%)		28 (6.0%)		2 (0.4%)		6 (1.3%)		2 (0.2%)	
**Talk about death (*n* = 465)**			**Yes, Since I Have No Problem and I Talk About it When I Want**		**Yes, But When It Happens to Me Closely**		**Sometimes, but with People from my Environment and Professional Profile**		**Seldom**		**Never**	
			208 (44.7%)		31 (6.7%)		97 (20.9%)		114 (24.5%)		15 (3.2%)	

* Without data specification (15). ** Without data specification (5).

**Table 3:** Topics received and interest in palliative care.

	Training Received in PC						Interest in PC Themes					
	Medicine *n* = 119		Psychology *n* = 76		Nursing *n* = 167		Academics *n* = 61			Total Students *n* = 383		
	YES	NO	YES	NO	YES	NO	High	Medium	Low	High	Medium	Low
General concepts	69 (58.0%)	50 (42.0%)	36 (47.4%)	40 (52.6%)	116 (69.5%)	51 (30.5%)	70.5%	27.9%	1.6%	80.9%	16.7%	2.3%
Rights, Policies	42 (35.3%)	77 (64.7%)	36 (47.4%)	40 (52.6%)	108 (64.7%)	59 (35.3%)	55.7%	36.1%	8.2%	70.5%	25.6%	3.9%
Oncological PC	28 (23.5%)	91 (76.5%)	9 (11.8%)	67 (88.2%)	84 (50.3%)	83 (49.7%)	54.1%	31.1%	14.8%	77.5%	17.5%	5.0%
Non-Oncological PCs	29 (24.4%)	90 (75.6%)	12 (15.8%)	64 (84.2%)	89 (53.3%)	78 (46.7%)	59.0%	32.8%	8.2%	75.2%	20.6%	4.2%
Pediatric PC	19 (16.0%)	100 (84.0%)	9 (11.8%)	67 (88.2%)	35 (21.0%)	132 (79.0%)	55.7%	29.5%	14.8%	80.4%	15.4%	4.2%
Needs people end of life	47 (39.5%)	72 (60.5%)	26 (34.2%)	50 (65.8%)	104 (62.3%)	63 (37.7%)	62.3%	32.8%	4.9%	80.2%	15.9%	3.9%
Physical symptoms	34 (28.6%)	85 (71.4%)	12 (15.8%)	64 (84.2%)	105 (62.9%)	62 (37.1%)	62.3%	27.9%	9.8%	83.8%	12.3%	3.9%
Nursing care	21 (17.6%)	98 (82.4%)	10 (13.2%)	66 (86.8%)	126 (75.4%)	41 (24.6%)	42.6%	27.9%	29.5%	66.3%	26.1%	7.6%
End of life emergencies	20 (16.8%)	99 (83.2%)	6 (7.9%)	70 (92.1%)	67 (40.1%)	100 (59.9%)	50.8%	31.1%	18.0%	80.7%	14.1%	5.2%
Last days	24 (20.2%)	95 (79.8%)	9 (11.8%)	67 (88.2%)	88 (52.7%)	79 (47.3%)	62.3%	29.5%	8.2%	76.2%	18.3%	5.5%
Death and mourning	41 (34.5%)	78 (65.5%)	26 (34.2%)	50 (65.8%)	109 (65.3%)	58 (34.7%)	70.5%	27.9%	1.6%	79.9%	14.1%	6.0%
Psychological aspects, communication	39 (32.8%)	80 (67.2%)	21 (27.6%)	55 (72.4%)	107 (64.1%)	60 (35.9%)	72.1%	26.2%	1.6%	79.9%	15.4%	4.7%
Social aspects	30 (25.2%)	89 (74.8%)	16 (21.1%)	60 (78.9%)	100 (59.9%)	67 (40.1%)	62.3%	34.4%	3.3%	72.3%	22.5%	5.2%
Cultural and spiritual aspects	29 (24.4%)	90 (75.6%)	16 (21.1%)	60 (78.9%)	95 (56.9%)	72 (43.1%)	66.9%	27.3%	5.8%	67.6%	26.6%	5.7%
Community aspects and networks	33 (27.7%)	86 (72.3%)	14 (18.4%)	62 (81.6%)	102 (61.1%)	65 (38.9%)	63.9%	32.8%	3.3%	70.5%	23.5%	6.0%
Social awareness	36 (30.3%)	83 (69.7%)	20 (26.3%)	56 (73.7%)	86 (51.5%)	81 (48.5%)	60.7%	36.1%	3.3%	70.2%	24.5%	5.2%
Volunteer programs	31 (26.1%)	88 (73.9%)	11 (14.5%)	65 (85.5%)	55 (32.9%)	112 (67.1%)	52.5%	39.3%	8.2%	74.2%	19.1%	6.8%
Integrated care	30 (25.2%)	89 (74.8%)	8 (10.5%)	68 (89.5%)	73 (43.7%)	94 (56.3%)	60.7%	34.4%	4.9%	69.5%	24.5%	6.0%
Tools to care	28 (23.5%)	91 (76.5%)	11 (14.5%)	65 (85.5%)	97 (58.1%)	70 (41.9%)	70.5%	24.6%	4.9%	79.4%	15.7%	5.0%
Compassionate, active listening, emotional	48 (40.3%)	71 (59.7%)	20 (26.3%)	56 (73.7%)	106 (63.5%)	61 (36.5%)	75.4%	23.0%	1.6%	84.3%	11.0%	4.7%
Investigation and evaluation	17 (14.3%)	102 (85.7%)	12 (15.8%)	64 (84.2%)	79 (47.3%)	88 (52.7%)	62.3%	29.5%	8.2%	77.5%	17.2%	5.2%
Management and organization	17 (14.3%)	102 (85.7%)	5 (6.6%)	71 (93.4%)	76 (45.5%)	91 (54.5%)	50.8%	41.0%	8.2%	71.3%	22.5%	6.3%
Networks and Compassionate Communities	20 (16.8%)	99 (83.2%)	8 (10.5%)	68 (89.5%)	67 (40.1%)	100 (59.9%)	55.7%	41.0%	3.3%	71.8%	23.0%	5.2%
Public politics	19 (16.0%)	100 (84.0%)	7 (9.2%)	69 (90.8%)	79 (47.3%)	88 (52.7%)	59.0%	34.4%	6.6%	67.9%	25.3%	6.8%
Rights of care	38 (31.9%)	81 (68.1%)	16 (21.1%)	60 (78.9%)	103 (61.7%)	64 (38.3%)	68.9%	29.5%	1.6%	80.7%	14.1%	5.2%

**Table 4:** Compassionate engagement and actions scale.

**Sex**	**Self-Compassion**			**Compassion for Others**			**Compassion from Others**		
	**Mean**	**Eng**	**Act**	**Mean**	**Eng**	**Act**	**Mean**	**Eng**	**Act**
Male	73.04	41.75	31.28	73	42.74	30.25	61.30	35.57	25.73
Female	69.67	39.91	29.75	72.47	42.03	30.44	60.18	34.71	25.46
	SELF-COMPASSION			COMPASSION FOR OTHER			COMPASSION FROM OTHERS		
				*t* = −0.356			*t* = −0.612		
				*p*-value = 0.722			*p*-value = 0.541		
**Age**	**Self-Compassion**			**Compassion for Others**			**Compassion from Others**		
	**Mean**	**Eng**	**Act**	**Mean**	**Eng**	**Act**	**Mean**	**Eng**	**Act**
18–39 years	70.32	40.19	30.13	72.29	41.98	30.30	60.46	34.86	25.59
40–59 years	71.71	41.69	30.02	74.53	43.81	30.71	59.83	35.12	24.71
> 60 years	79.75	45.5	34.25	81.75	46.25	35.5	69.75	40	29.75
	SELF-COMPASSION			COMPASSION FOR OTHER			COMPASSION FROM OTHERS		
	F = 1.012			F = 2.138			F = 1.331		
	*p*-value = 0.039 *			*p*-value = 0.119			*p*-value = 0.265		
	**Self-Compassion**			**Compassion for Others**			**Compassion from Others**		
	**Mean**	**Engagement**	**Action**	**Mean**	**Engagement**	**Action**	**Mean**	**Engagement**	**Action**
Academics	73.77	43.14	30.62	72.93	43.09	29.83	59.01	34.42	24.59
Medicine Student	69.03	39.23	29.79	72.36	42.10	30.25	59.95	34.04	25.91
Psychology Student	71.59	40.23	31.35	74.94	43.42	31.52	58.89	33.98	24.90
Nursing Student	69.41	39.86	29.55	71.29	41.08	30.20	61.82	35.92	25.89
	Self-Compassion			Compassion for Other			Compassion from Others		
	F = 3.327			F = 1.423			F = 0.607		
	*p*-value = 0.364 *			*p*-value = 0.242			*p*-value = 0.545		

* statistically significant.

## Data Availability

Data are available from Unisanitas data sources.
